# Effect of written outcome information on attitude of perinatal healthcare professionals at the limit of viability: a randomized study

**DOI:** 10.1186/s12910-019-0413-7

**Published:** 2019-10-22

**Authors:** V. Papadimitriou, B. Tosello, R. Pfister

**Affiliations:** 1 Neonatal and Paediatric Intensive Care Unit, University Hospitals of Geneva, and Geneva University, 1211 Genève, Switzerland; 2 Aix-Marseille Université, CNRS, EFS, ADES, Marseille, France

**Keywords:** Counselling, Attitudes, Survival, Resuscitation, Extremely preterm infant, Limit of viability

## Abstract

**Background:**

Differences in perception and potential disagreements between parents and professionals regarding the attitude for resuscitation at the limit of viability are common. This study evaluated in healthcare professionals whether the decision to resuscitate at the limit of viability (intensive care versus comfort care) are influenced by the way information on incurred risks is given or received.

**Methods:**

This is a prospective randomized controlled study. This study evaluated the attitude of healthcare professionals by testing the effect of information given through graphic fact sheets formulated either optimistically or pessimistically. The written educational fact sheet included three graphical presentations of survival and complication/morbidity by gestational age. The questionnaire was submitted over a period of 4 months to 5 and 6-year medical students from the Geneva University as well as physicians and nurses of the neonatal unit at the University Hospitals of Geneva. Our sample included 102 healthcare professionals.

**Results:**

Forty-nine responders (48%) were students (response rate of 33.1%), 32 (31%) paediatricians (response rate of 91.4%) and 21 (20%) nurses in NICU (response rate of 50%). The received risk tended to be more severe in both groups compared to the graphically presented facts and current guidelines, although optimistic representation favoured the perception of “survival without disability” at 23 to 25 weeks. Therapeutic attitudes did not differ between groups, but healthcare professionals with children were more restrained and students more aggressive at very low gestational ages.

**Conclusion:**

Written information on mortality and morbidity given to healthcare professionals in graphic form encourages them to overestimate the risk. However, perception in healthcare staff may not be directly transferable to parental perception during counselling as the later are usually naïve to the data received. This parental information are always communicated in ways that subtly shape the decisions that follow.

## Background

Children born at extremely low gestational ages represent 0.3% of all live births [1]. Improved perinatal intensive care has increased survival but is associated with neurological handicap amongst survivors [2–5]. In numerous countries, guidelines recommend basing resuscitation effort practices on informed parental decision before birth from individual obstetric information [6–8]. The Fetus and Newborn Committees for the USA and Canada advocate informed decision-making at the limits of neonatal viability and respect for parental autonomy [7–9]. For situations at the limit of viability, professional recommendations of the Swiss Society of Neonatology put an active resuscitation in balance with the suffering of intensive care and expected quality of life [10, 11]. It appears reasonable to provide supportive instead of intensive care to limit suffering, if this equation is individually, socially and sometimes even financially out of balance.

Prenatally, gestational age is preferentially used to make a resuscitation decision, although several other parameters need to be taken into account. Indeed, gender, fetal weight, number of fetuses and completed lung maturation are known factors that influence the child’s prognosis and have led to prognostic computations (like this tool: *National Institute of Child Health and Human Development Neonatal Research Network*) [8]. Parents find it useful to create general guidelines for physicians and simplify parental information [8]. Probably, the detailed presentation could reduce parental anxiety and increase their knowledge of long-term problems related to prematurity [12]. Swiss recommendations define a « grey zone » in which, in addition to gestational age, the cited hard criteria guide the attitude [10]. In short, for a gestational age less than to 24 weeks it is recommended to limit the attitude to comfort care unless there are favourable factors and strong parental demand. Some authors argue that the use of guidelines is irrational [13]. Most tools or guidelines use gestational age to guide counseling, however it is ethically questionable and illogical to base decisions concerning the care of infants at the limits of viability on a gestational age label [13].

Then, how can a shared decision policy for parents be implemented? While tools such as decision aids are meant to facilitate decision-making; these are sometimes perceived by professionals as too general, not taking into account the individuality of the situation.

Miscommunication is a frequent occurrence, and may in part arise from providers’ insufficient (or inadequate) knowledge of outcomes of extremely low birth weight infants [14]. The survival rates for infants < 27 weeks gestation were underestimated and disability rates were overestimated by 10–50% compared to published data [14]. A study in extremely premature neonates showed for instance, that healthcare professionals, often criticised for supporting too aggressive treatment, are in fact more inclined to limit treatment in unfavourable conditions than parents [15]. It has also been reported that professional experience influences this attitude, but a better understanding of what influences the decision-making process and attitudes in the health care team would be of great help [16].

There is evidence that healthcare professionals’ training could play an important role. Mehotra et al. found significant deficiencies across US NICUs with neonatology training programs [17]. In addition, Boos et al. showed parents have difficulties understanding resuscitation options, and furthermore they do not recall discussing these options with their clinicians [18]. For example, some parents feel that they do not participate in decision-making at the time of birth, whereas clinicians believe the opposite [18].

We hypothesized that healthcare decisions concerning resuscitation of newborns at the limit of viability (intensive care versus comfort care) are influenced by the way information on incurred risks is given or received.

## Methods

This is a prospective study on ethical decision-making and attitudes of physicians, nurses and medical students facing preterm neonates at the limit of viability. It analyses the therapeutic attitudes in different situations below 28 weeks postmenstrual age through a randomized prospective design testing the effect of a fact sheets formulated either in an optimistic (probability to be healthy) or pessimistic (risks of death or morbidity) presentation.

The study protocol had been submitted to the Ethics Committee of the University Hospitals of Geneva, which considered that a formal authorization was not necessary, as the questionnaire concerned staff who were free to answer or not, and no patients were directly affected.

### Population

The questionnaire was submitted over a period of 4 months to 5th and 6th-year medical students from the Geneva University (*n* = 148), physicians (paediatricians and neonatologists, *n* = 35) and nurses of the neonatal unit at the University Hospitals of Geneva (HUG) (*n* = 42). The only non-inclusion criterion was the refusal to participate in the study. The professionals had accepted the study’s participation principles and signing an informed consent.

### Randomisation

The randomisation was carried out by random allocation of an information sheet, either Op sheet (optimistic) or Pe sheet (pessimistic). Op and Pe sheets were mixed in blocks of 10, each one containing 10 Op or 10 Pe information sheets. Each practitioner was randomly assigned either an Op or Pe sheet until the block was completely full, then randomisation began among a new block. Participants were asked to carefully read the randomly assigned fact sheet before filling out a questionnaire on perception and attitude, but were encouraged not to consult the former between each question.

### Fact sheet

Two types of fact sheets, differing only in layout but not (in) its underlying content, were randomly attributed to the population at test. Written educational fact sheet included three graphical depictions of survival and complication/morbidity according to gestational age. Morbidities were limited to intracranial haemorrhage, bronchopulmonary dysplasia, periventricular leucomalacia, necrotizing enterocolitis and severe disability (defined as psychomotor or mental retardation, bilateral blindness or deafness, or cerebral palsy). Data reported in the Swiss recommendations was used to generate the graphs [10]. Responses were analyzed according to the NICHD Neonatal Research Network data by prognostic indicators [8].

In the optimistic group (Op), the fact sheet representation was ‘optimistic’ or positive: chances of survival, absence of complications or severe disability. In the classic or pessimistic group on the other hand (Pe), it rather had a classic presentation in medical practice, i.e. it put forward the negative aspects: risk of either mortality or complications and disabilities.

### Survey questionnaire

Clear instructions and study issues were presented on a cover page. At any time, the participant had the choice of waiving the continuation of the questionnaire. The survey was anonymous and required approximately 10 min to complete.

The questionnaire was structured in two parts. The first section was dedicated to demographics, including questions regarding factors influencing the ethical attitude reported by others [1] such as age, gender, nationality, religion, professional experience, marital status, number of children and personal experience with a premature child.

The second section presented five clinical vignettes, each representing a different case of extreme prematurity. The fictitious stories were prepared with a grading according to the risk at gestational ages between 23 and 27 weeks, including weight, sex and presence or absence of lung maturation. However, vignettes were administered randomly related to the risk and followed by two sets of questions:

The first set of questions concerned the **perception of risks**. A choice was given between four possible outcomes was given: death, profound disability, severe disability, or survival without disability or with mild impairment. For each issue, a probability scale from 0 to 100% was proposed.

The second set of questions concerned the **therapeutic attitude**. Four choices were proposed: 1. No resuscitation and comfort care only; 2. Comfort care, unless the child is vigorous at birth; 3. Intensive care by principle, unless the child is depressed or not vigorous and; 4. Maximal resuscitation efforts and maximum intensive care.

Fact sheet and survey questionnaire are detailed in the Additional file 1.

### Statistical analysis

The sample size for a 10% difference between the groups Op and Pe, with a power of 80% and a risk of type I error of 0.05, was roughly estimated to require 100 subjects. For this calculation the vignette with the smallest expected difference was chosen (27 weeks of gestation), therefore supposed to be the most critical in statistical terms. The sample size of 100 was confirmed after a pilot of a dozen questionnaires based on the Altman nomogram [19].

Socio-demographic characteristics were analysed according to standard descriptive statistics (frequency, mean, standard deviation) and quantitative variables were tested using Chi-Square, while the Student’s T-test was used for continuous variables. The Mann-Whitney U test was used for qualitative variables. ANOVA with a post-hoc test was used to compare the difference between individual professional groups. Computation was performed with SPSS (PASW statistics 18, release 18.0.0, Jul 30, 2009).

## Results

In this randomized, controlled trial, 102 participants responded to the questionnaire, 63% (65) were women, 65% (66) Christians and 23.5% (24) nonreligious. The participants’ countries of origin were mainly Switzerland (62.6%; 64) and France (22.5%; 23).

Forty-nine responders (48%) were students (response rate of 33.1%), 31% (32) paediatricians (response rate of 91.4%) and 20% (21) nurses working with newborns (response rate of 50%). Twenty-six (25%) participants were married and 32.3% (33) were parents with an average of 2 children per family.

The overall rate of missing values was 1–3% distributed evenly throughout the vignettes.

For the perception of risk, a significant difference was observed between groups Op and Pe for the item “survival without disability” at low gestational ages (23 weeks: 17.6% (18) vs 7.8% (8), *p* = 0.013; 24 weeks: 27.4% (28) vs 17.6% (18), *p* = 0.023 and 25 weeks: 35.2% (36) vs 25.4% (26), *p* = 0.018; 26 weeks: 43.1% (44) vs 46% (47), *p* = 0.60). Perceived risk in both groups significantly diverged from the presented figures of the Swiss guidelines (Table 1) except for “survival without disability” when the information was positive.

**Table 1:** Risk perception according to type of information compared to the Swiss guidelines

GA (wks)	Outcome	Reference [9]	Optimistic information, mean (+/−SD)	*P* value*	Pessimistic information, mean (+/−SD)	*P* value**
23	Death	0.68	0.87 (0.15)	< 0.001	0.90 (0.08)	< 0.001
	Profound impairment	0.39	0.77 (0.20)	< 0.001	0.73 (0.21)	< 0.001
	Severe impairment	0.39	0.35 (0.32)	NS	0.31 (0.31)	NS
	Without profound or severe disability	0.31	0.17 (0.20)	< 0.001	0.08 (0.12)	< 0.001
24	Death	0.40	0.71 (0.12)	< 0.001	0.73 (0.13)	< 0.001
	Profound impairment	0.31	0.57 (0.24)	< 0.001	0.57 (0.22)	< 0.001
	Severe impairment	0.33	0.46 (0.28)	0.002	0.38 (0.27)	NS
	Without profound or severe disability	0.35	0.27 (0.24)	0.03	0.17 (0.16)	< 0.001
25	Death	0.24	0.50 (0.11)	< 0.001	0.54 (0.08)	< 0.001
	Profound impairment	0.21	0.45 (0.24)	< 0.001	0.40 (0.20)	< 0.001
	Severe impairment	0.24	0.51 (0.22)	< 0.001	0.43 (0.22)	< 0.001
	Without profound or severe disability	0.54	0.35 (0.23)	< 0.001	0.25 (0.18)	< 0.001

[9] Swiss Society of Neonatology, Berger, TM et al., Swiss Med Wkly 2011;141:w13280

*Significant difference between Swiss recommendations (reference) and optimistic information. ** Significant difference between Swiss recommendations (reference) and pessimistic information

The risk perception of mortality in both groups significantly diverged at 25 weeks compared to NICHD Neonatal Research Network calculator (Table 2).

**Table 2:** Risk perception of mortality compared with NICHD Neonatal Research Network calculator

GA (wks)	NICHD calculator	Optimistic information, mean (+/−SD)	*P* value*	Pessimistic information, mean (+/− SD)	*P* value**
23	89%	0.87 (0.15)	0.545	0.90 (0.08)	0.218
24	70%	0.71 (0.12)	0.306	0.73 (0.13)	0.049
25	15%	0.50 (0.11)	< 0.001	0.54 (0.08)	< 0.001

*Significant difference between “NICHD Neonatal Research Network calculator” [3] and optimistic information. **Significant difference between “NICHD Neonatal Research Network calculator” and pessimistic information

For the therapeutic attitude, there was no statistically significant difference between the groups Op and Pe with the Mann-Whitney U test (Table 3), even when forcing the attitude into a binary comparison. A gradual increase in decisions to maximal resuscitation was observed with increasing gestational age; 22.5% (23) of participants at 23 weeks and 94.1% (96) at 27 weeks favoured maximal resuscitation (Table 3).

**Table 3:** Attitudes of maximal resuscitation according to the type of information

GA (wks)	Total % (n)	Optimistic information % (n)	Pessimistic information % (n)	*P* Value
23	22.5% (23/102)	16% (8)	31% (15)	0.09
24	49% (50/102)	46% (24)	53% (26)	0.69
25	78% (80/102)	82% (42)	78% (38)	0.33
26	90% (92/102)	98% (50)	88% (42)	0.01
27	94% (96/102)	98% (50)	94% (46)	0.09

**Fig. 1:**
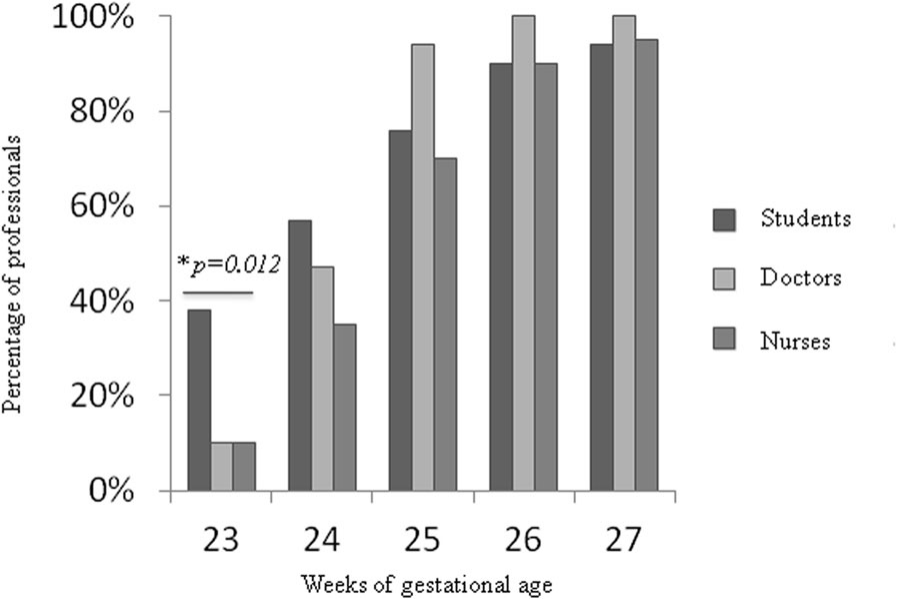
Attitudes of maximal resuscitation by weekly strata according to professional group

The differences between occupational groups were statistically significant at 23 weeks only (ANOVA, *p* = 0.011); a majority (90.1%; 92) of the medical and nursing staff choose comfort care compared to 62% (30) of the students. At 25 weeks, 12.5% (4) of physicians, 23.8% (5) nurses and 18.4% (9) students still preferred comfort care. Attitudes of maximal resuscitation by weekly strata and professional group are reported in Fig. 1.

**Fig. 2:**
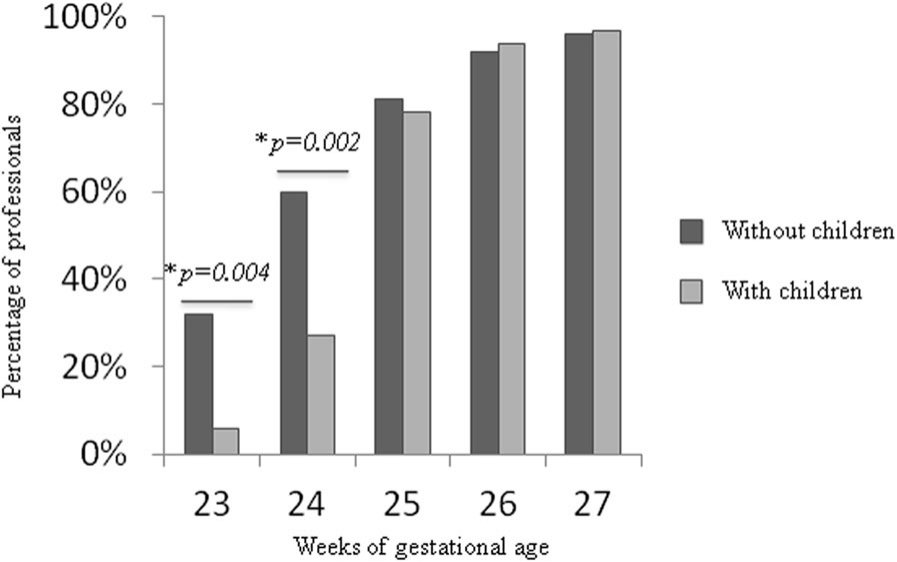
Attitudes of maximal resuscitation by weekly strata according to the staff’s family status

There was a significant difference in therapeutic attitude at 23 and 24 weeks between healthcare staff with and without children (Fig. 2), but no differences for gender or religious beliefs.

## Discussion

This work suggests that written graphic information tends to lead to an overestimation of the risk perception in health care staff, although an ‘optimistic’ presentation may attenuate this perception. Decision-making and therapeutic attitudes varied significantly between occupational and social groups but remained overall close to the provided information and actual guidelines. The presented data corroborates previous studies that found an influence of personal experience on decision-making at the limit of viability [20].

The results showed a strong consensus towards maximal resuscitation from 26 weeks on, while disparities remained between 23 and 25 weeks, confirming the current dilemma or grey-zone regarding resuscitation of extremely preterm neonates at birth in Switzerland but also elsewhere [21].

While there is no identical approach on the management at the limit of viability amongst institutions [11], Switzerland has pioneered in 2006 [22] by publishing one of the first ethical guidelines at the limit of viability that defined a grey-zone of decision-making with parents for local and personal use in the NICU. As guidelines influence practice, so may practice influence guidelines and it becomes difficult to interpret the origins of nurses’ and physicians’ attitudes. For students however, who were naïve to those recommendations, the therapeutic attitude appears almost entirely driven by social norms and values.

A Cochrane review found that when facing health choices or screening options, decision aids led to significantly improved knowledge and less passive decision-making [23]. However, unlike the study by Haward et al., where the presentation of information in positive form positively influenced parental decision-making, the findings on therapeutic attitude of healthcare staff did not confirm this effect in a randomized controlled design, although the risk perception was influenced and there was a trend favouring resuscitation at higher gestational ages [24]. This may be explained by the fact that healthcare workers, especially physicians, were already knowledgeable of figures and therefore less influenced by this type of presented information. Also, the fragile emotional state of parents may be more likely to accept guidance by an ‘optimistic’ or ‘pessimistic’ presentation compared to more emotionally detached healthcare professionals. Finally, the very short interval between the reading of the fact sheet and the filling of the questionnaire may allow less time for an emotional risk perception. The recent literature showed that theoretical and empirical evidence for the use of framing strategies could reduce the development of nocebo side effects with the rationale that positively framing such information could diminish this risk [25, 26]. Other authors suggest that the studies using multiple modes (including video, which comprises visual and verbal presentation methods) elicited numerically larger framing effect sizes, suggesting that multimodal presentation may more successfully elicit the framing effect [27, 28].

Other studies have found physicians and nurses tend to considerably underestimate survival and overestimate major neurodevelopmental disability, even shortly after reading the facts [29]. Overall, nurses working in neonatology were the least supportive group of maximal resuscitation at any of the gestational ages tested: they remained mostly in line with physicians for attitudes in premature infant below 25 weeks.

A potential limitation of this study was the risk of bias due to a population composed of distinct occupational groups with very divergent personal and professional experience. Moreover, more than a punctual decision of “resuscitation or not” at birth, a complete prenatal approach (including antenatal steroids, mode of delivery in particular), and postnatal approach after the delivery room, and repeated ethical questioning over the whole perinatal period are at stake.

Nevertheless, this multidisciplinary aspect of the cohort reflects the everyday reality when dealing with extreme prematurity. The student population added a naïve group in terms of practice and experience, thus revealing some aspects of professional developmental in decision-making. During their studies, specific ethical questions on neonatal care at the limit of viability are not part of the curriculum. Students with little or no clinical experience were significantly more prone to maximal resuscitation at 23 and 24 weeks of gestation than clinicians, whether physicians or nurses. Thus from the results, one may deduce that increasing personal experience and close relation with the patient tends to reduce aggressive intervention.

Physicians responding to the questionnaire were generally more exposed to situations of therapeutic dilemmas at the limit of viability and would thus more easily link the survival curves of the fact sheet to current resuscitation practices and burden of care to be endured by newborns, in addition to the realities of long-term follow-up and quality of life [30]. Physicians became more inclined to full resuscitations from 25 weeks on, whereas nurses remained hesitant. This generally reserved nursing attitude at the limit of viability has already been reported before and may be explained by the more intimate and personal experience of continued and direct contact with the critical patients [31].

Behaviour at the limit of viability may be explained by arguments that are not primarily medical but normative instead. The moral obligation to offer the newborn a life worth living is socially influenced by priorities given towards a ‘superior interest’ and carried by parental responsibility [32]. It ultimately reflects the fact that parents follow a social pattern by accepting that the decision to limit or withdraw life-sustaining support is made in the majority of countries by the healthcare team despite their profound concern in the decision-making [32].

In general, the literature reports diverging processes in ethical decision-making between men and women [33], a finding found in the present study. The religious affiliation did not influence the decision making in our study [34]. However, the healthcare staff with children of their own were less inclined than those without children to give full resuscitation, particularly at lowest gestational ages. This could reflect the strong feeling of attachment and protection developed towards the own child and substituted into the critical case. The personal situation becomes a direct social reference for the expected long-term risk of resuscitation.

A hypothesis is that differences in decision-making between obstetricians and neonatologists will influence prenatal care [35] and parental counselling, and may be the cause of some inter-professional conflicts. Such disagreement has even been shown to alter neonatal mortality [36]. Furthermore, parents express a need for a coherent message in situations at the limit of viability [37], so that a joint prenatal delivery plan based on a consensual target is highly desirable [38, 39]. These data may help understand and support a multidisciplinary process by showing that written graphical information influences understanding and the perception of risk, but that decision-making for a therapeutic attitude significantly depend on social and personal factors, and experience, be it on the parents’ or healthcare staff’s side. As these socially imprinted attitudes are difficult to influence, a grey zone of ethical ambiguity is unmasked. It appears obvious and is increasingly recognized that a single person counselling does not take into account the full complexity of a situation expressed in this grey zone [39]. Advocating for a multidisciplinary healthcare team to reach a therapeutic grey zone consensus is fundamental, so should an objective parental counseling also be done jointly by representatives of the obstetric and neonatal team. Until recently, doctors saw their primary role as providing parents with detailed, objective information about treatment choices and likely outcomes. A newer approach recommends that doctors help parents discern their own values and ethical commitments as they face an unanticipated situation and a series of lifealtering decisions [40]. The shift of focus from result to process is subtle but important; instead of an ethics of conflict resolution, this approach requires an ethics of value clarification [41].

The power of this study has been confirmed for the situation with the smallest statistical difference at 27 weeks, but the subgroup analysis between the three professional groups is limited. A further critique to the study design was the limited graphical illustration of the main complications in prematurity in order to keep the information sheet short and pragmatic. The vignettes of extremely preterm infants were not detailed in terms of long-term outcome and may thus have biased the interpretation of more expert participants. However, a short vignette like the ones created, cannot take into account the broad information of real life on which most decisions depend despite incomplete prenatal information being common when time for detailed decision-making is scarce. A graphic fact sheet may therefore have the advantage of a fast delivery of information. As the risk perception is often overestimated at the youngest gestational ages, an ‘optimistic’ presentation would tend to favor a realistic perception, apparently without adverse effect on attitude.

## Conclusion

Written information on mortality and morbidity given to healthcare professionals in graphic form on a fact sheet, whether positive or negative overall encouraged some professionals to overestimate the risk, especially at lowest gestational ages and more so for the negative pessimistic information. However, perception in healthcare staff may not be directly transferable to parental perception during counselling as the later are usually naïve to the prognostic data received. Shared decision making aims to enable patients “to take an active role in deciding about and planning their health care” [42]. Although shared decision making is the ethically preferred approach [43], but most neonatologists do not share decision making well. According to John Lantos [41], “*facts do not speak for themselves. They are always and inevitably communicated in ways that subtly shape how people interpret them and thus subtly shape the decisions that follow*… *Autonomy is realized and exercised only through caring relationships; people need the help of others to act autonomously.*”

## Supplementary information

**Additional file 1: Appendix 1.** Introductory letter, Information sheets A (Optimistic sheet) and B (Pessimistic sheet) and questionnaire with clinical cases (translated from French).

## Data Availability

The datasets used and /or analysed during the current study are available from the corresponding author on reasonable request.
