# Supplementary material for: Effect of written outcome information on attitude of perinatal healthcare professionals at the limit of viability: a randomized study
Source: BMC Med Ethics. 2019 Oct 22;20:74. doi: 10.1186/s12910-019-0413-7 (PMC6806555; doi:10.1186/s12910-019-0413-7)
Supplement: Supplementary file 1 — Additional file 1: Appendix 1. Introductory letter, Information sheets A (Optimistic sheet) and B (Pessimistic sheet) and questionnaire with clinical cases (translated from French). [file 12910_2019_413_MOESM1_ESM.pdf]

Appendix 1: Introductory letter, Information sheets A (Optimistic sheet) and B (Pessimistic sheet) and questionnaire with clinical cases (translated from French)

Dear participant, Ladies and Gentlemen,

The number of premature children surviving at an increasingly younger gestational age is on a constant rise.

However, intensive care is burdensome and outcomes are not always favorable, thus raising legal and moral questions concerning the decision-making for the management at the limit of viability.

This study aims to evaluate how specific information may influence the decision-making for critical cases born at the limit of viability. If we found that different presentations of the same risk influence the therapeutic attitude, such finding would challenge the objectivity of the information given.

The current evaluation is based on an anonymous survey that will take around ten minutes of your time, and is submitted during 6 months to approximately 100 volunteers in the medical profession (physicians, nurses and medical students), but no to parents or patients.

We would be very grateful for a sincere answer. Your free participation is very much appreciated. If you do not want to participate to the study, do not return the questionnaire. By participating, you will support a scientific project and you agree to the anonymous and confidential use of the given information.

Thank you for your collaboration,

Information A

Survival according to gestational age (postmenstrual age in weeks)

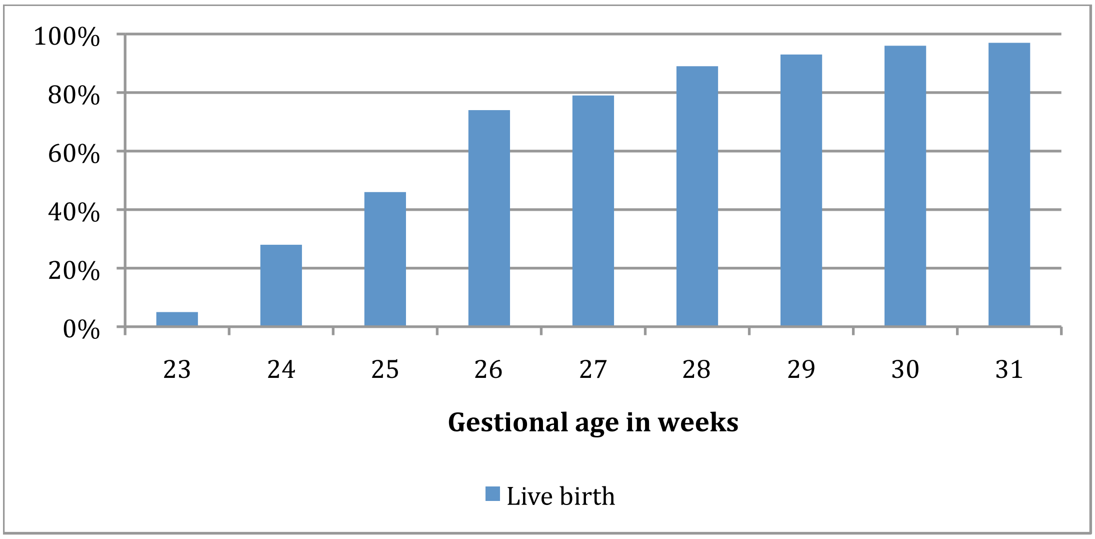

Free of major complications according to gestational age. Complications mainly include intracranial hemorrhage, bronchopulmonary dysplasia, periventricular cystic leucomalacia and necrotizing enterocolitis.

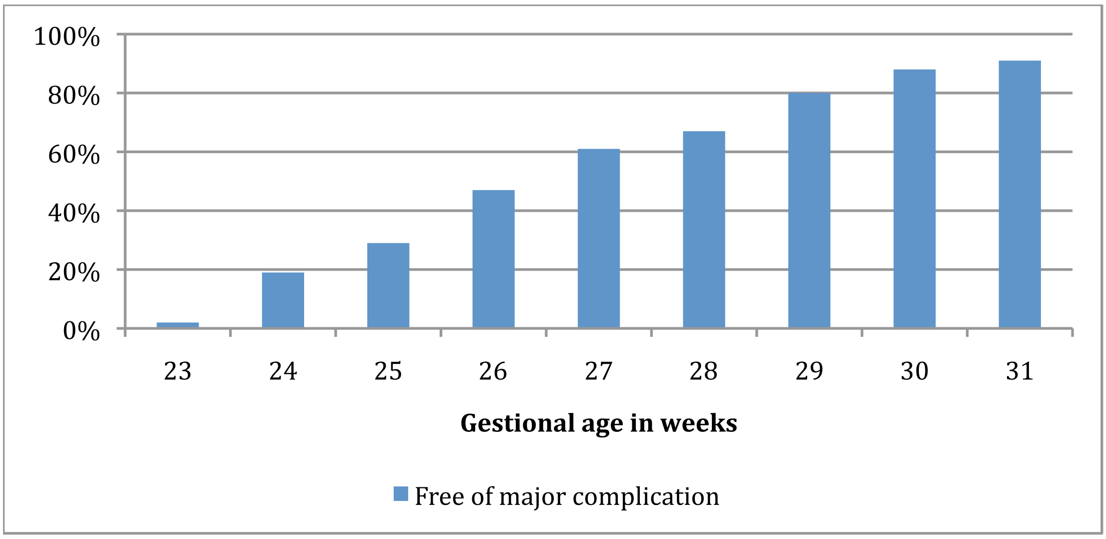

Survival without severe handicap (cognitive or psychomotor: bilateral blindness, bilateral loss of auditive function, cerebral palsy)

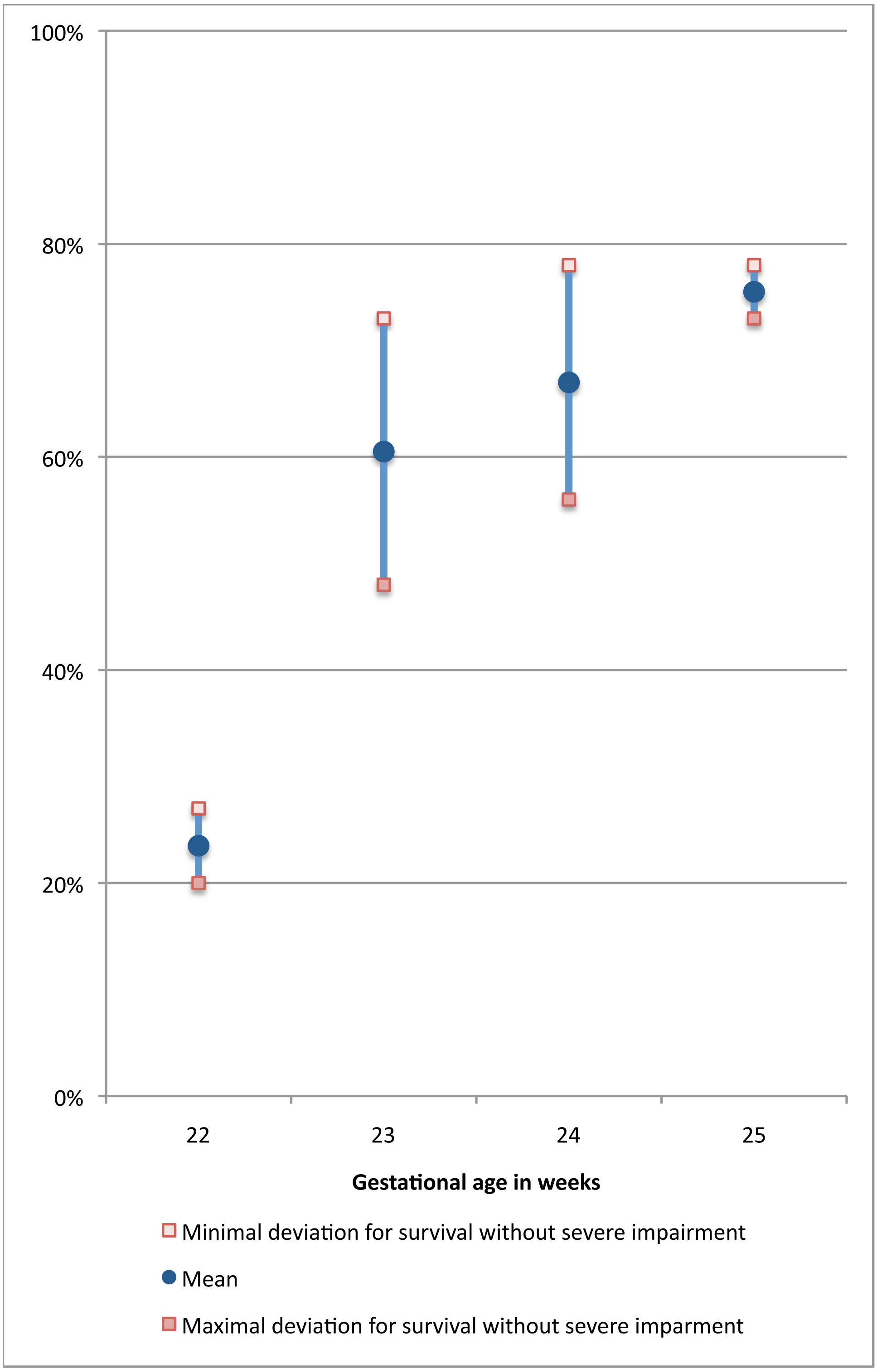
100%
80%
60%
40%
20%
0%
22
23
24
25
Gestational age in weeks
Minimal deviation for survival without severe impairment
Mean
Maximal deviation for survival without severe imparment

Information B

Mortality of the newborn according to gestational age (postmenstrual age in weeks)

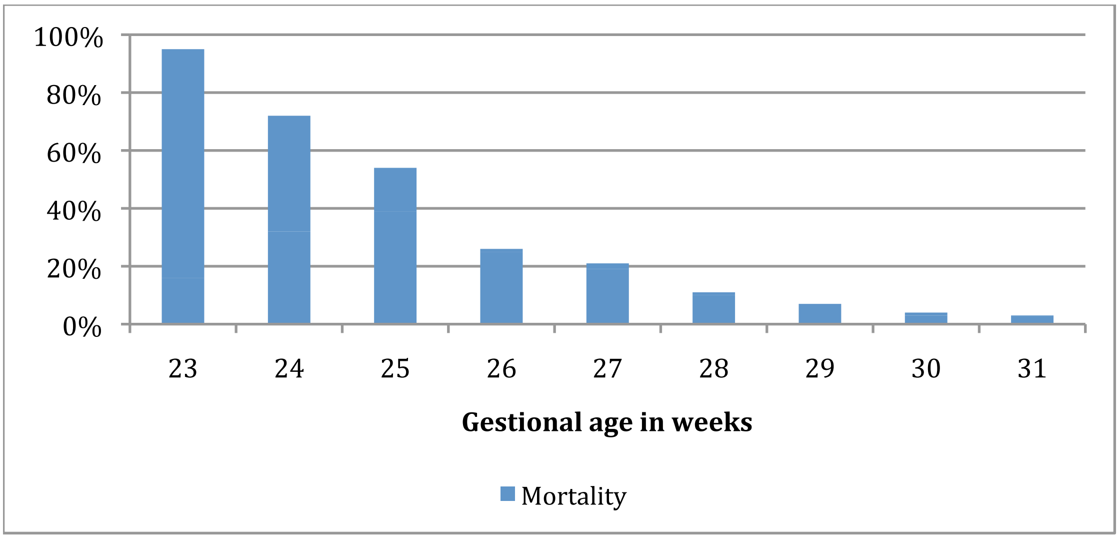

Major complications of the premature newborn according to gestational age. These complications mainly include intracranial hemorrhage, bronchopulmonary dysplasia, periventricular cystic leucomalacia and necrotizing enterocolitis.

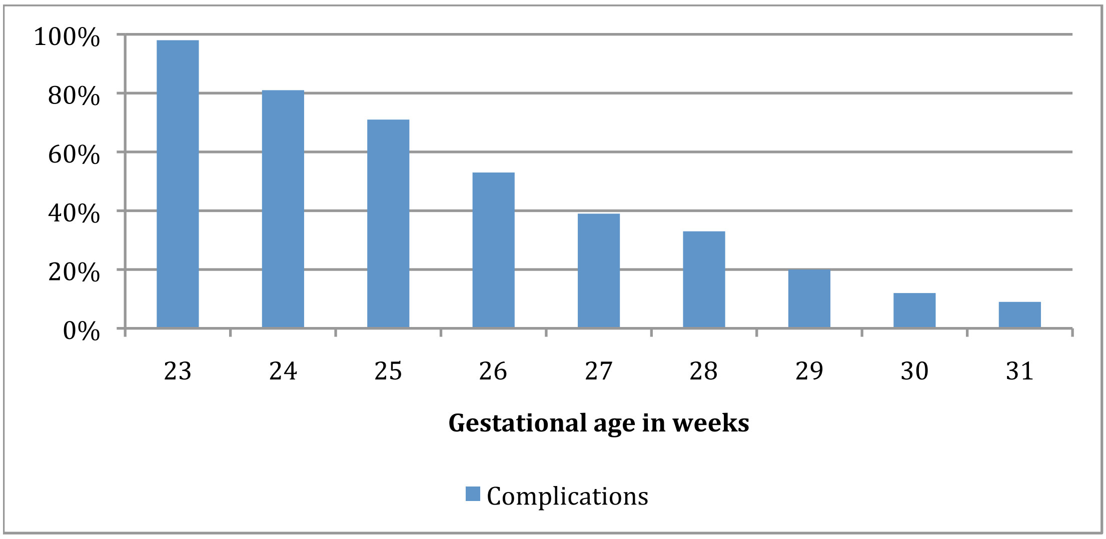

Risk of survival with severe handicap (cognitive or psychomotor: bilateral blindness, bilateral loss of auditive function, cerebral palsy).

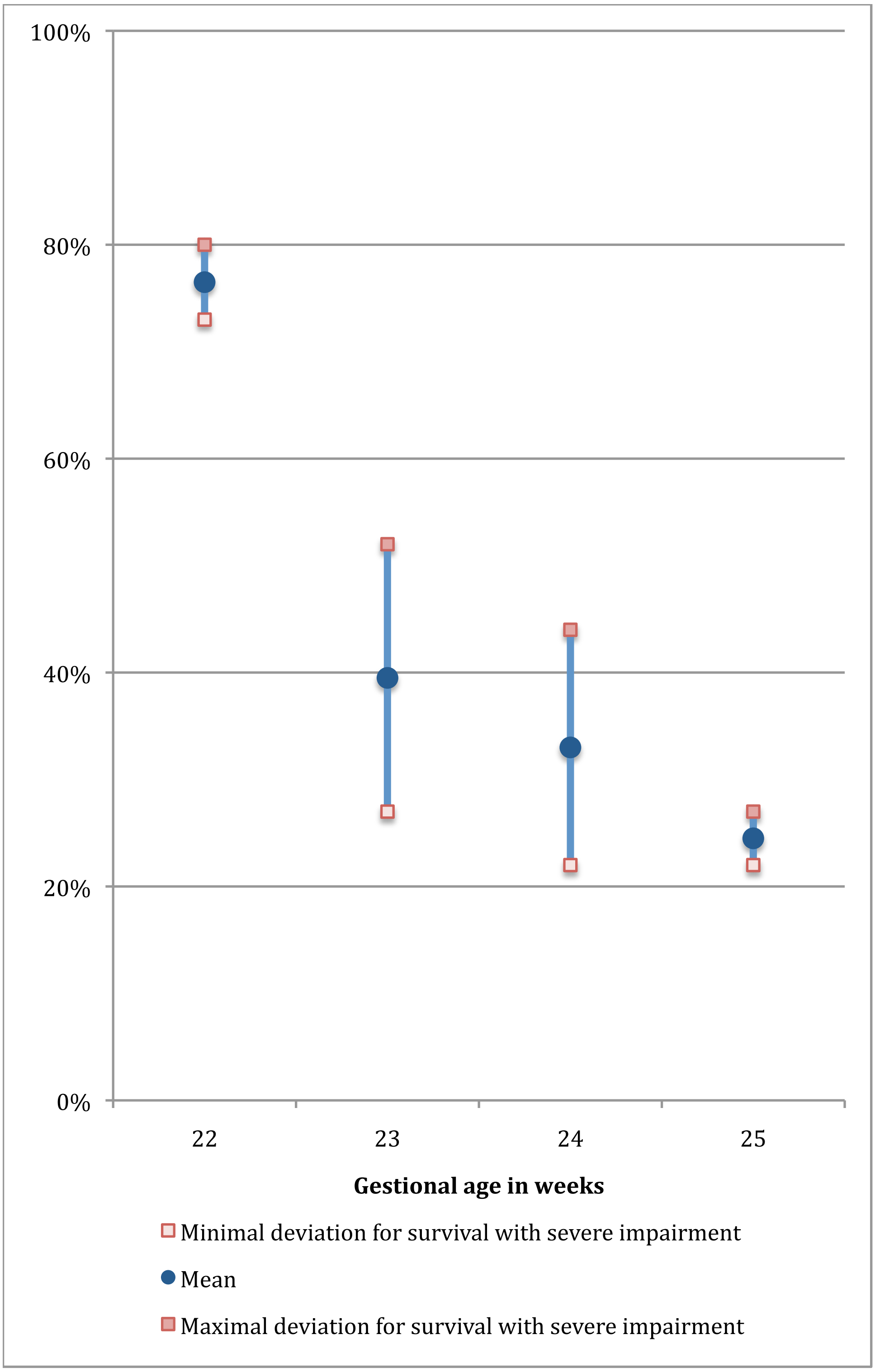
100%
80%
60%
40%
20%
0%
22
23
24
25
Gestional age in weeks
Minimal deviation for survival with severe impairment
Mean
Maximal deviation for survival with severe impairment

Clinical cases

Did you receive information sheet □ A or □ B ?

Case 1

The mother is 29 year old and at 25 0/7 weeks, hospitalized since 24 hours due to uterine contractions. Steroids for lung maturation were immediately initiated at admission. Despite bedrest and tocolitics, contractions persist and delivery becomes necessary per cesarean section after finalized pulmonary maturation (due to previous cesarean section and pain localized in the scar). A girl is expected with an estimated weight of 750 gr.

1) What is your estimated risk?

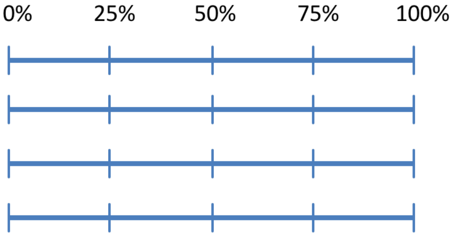

  i. Death of child
  ii. Very severe handicap
  iii. Survival with severe handicap
  iv. Survival without profound or severe handicap

2) What is your therapeutic decision?
  i. No resuscitation and comfort care only
  ii. Comfort care, unless the child is vigorous at birth
  iii. Intensive care in principle, unless the child is depressed or non vigorous
  iv. Maximal resuscitation and maximal intensive care

Case 2

The mother is 40 year old, and at 23 1/7 weeks, consults her gynecologist because of pelvic pain since 48 hours. After investigations, a chorioamnionitis (infection of membranes and of amniotic liquid) is diagnosed that needs an extraction of the child. A boy is expected with an estimated weight of 510 gr.

1) What is your estimated risk?

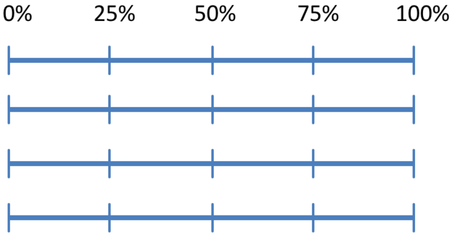

  i. Death of child
  ii. Very severe handicap
  iii. Survival with severe handicap
  v. Survival without profound or severe handicap

2) What is your therapeutic decision?
  i. No resuscitation and comfort care only
  ii. Comfort care, unless the child is vigorous at birth

iii. Intensive care in principle, unless the child is depressed or non vigorous
iv. Maximal resuscitation and maximal intensive care

Case 3

The mother is 32 year old and at 26 1/7 weeks. She arrives at the gynecological emergencies for regular uterine contractions. A full course of antenatal steroids can be given. A girl is expected with an estimated weight of 790 gr.

1) What is your estimated risk?
   i. Death of child
   ii. Very severe handicap
   iii. Survival with severe handicap
   vi. Survival without profound or severe handicap

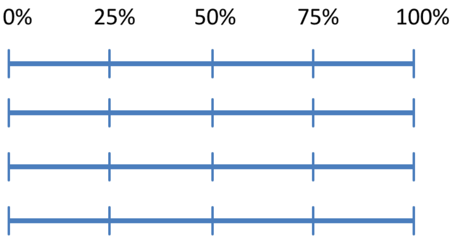

2) What is your therapeutic decision?
   i. No resuscitation and comfort care only
   ii. Comfort care, unless the child is vigorous at birth
   iii. Intensive care in principle, unless the child is depressed or non vigorous
   iv. Maximal resuscitation and maximal intensive care

Case 4

The mother is 38 year old and at 27 5/7 weeks. She ruptured membranes during a consultation for flu-like symptoms (subfebrile at 37.6°C). There are twins with an estimated weights of 1000gr each.

1) What is your estimated risk?
   i. Death of child
   ii. Very severe handicap
   iii. Survival with severe handicap
   vii. Survival without profound or severe handicap

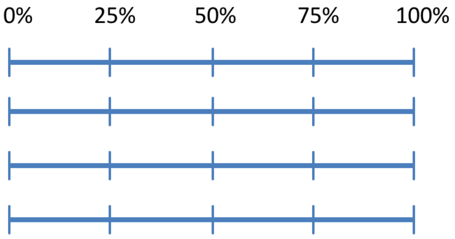

2) What is your therapeutic decision?
   i. No resuscitation and comfort care only
   ii. Comfort care, unless the child is vigorous at birth
   iii. Intensive care in principle, unless the child is depressed or non vigorous
   iv. Maximal resuscitation and maximal intensive care

Case 5

The mother is 25 year old and at 24 0/7 weeks. She comes to the emergency station after rupture of membranes. No antenatal steroids have been given. A boy is expected with an estimated weight of 600 gr.

1) What is your estimated risk?
   i. Death of child
   ii. Very severe handicap
   iii. Survival with severe handicap
   viii. Survival without profound or severe handicap

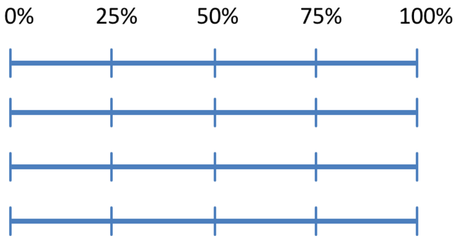

2) What is your therapeutic decision?
   i. No resuscitation and comfort care only
   ii. Comfort care, unless the child is vigorous at birth
   iii. Intensive care in principle, unless the child is depressed or non vigorous
   iv. Maximal resuscitation and maximal intensive care
